# Personal

**Published:** 1905-09

**Authors:** 


Dr. E. L. Shurly, of Detroit, was elected president of the American Climatological Association at its recent annual meeting held in that city. Dr. Shurly is also editor of the new journal of tuberculosis.

---

Dr. Frank P. Foster, editor of the New York *Medical Journal,* was elected a vice-president of the Medical Editors' Association at its recent annual meeting held at Portland, Oregon.

---

Dr. Edgar R. McGuire, of Buffalo, is visiting Europe in company with Dr. Roswell Park. After a tour of Great Britain he will attend the International Surgical Congress at Brussels, then visit Paris, returning to Buffalo about the middle of October.

---

Dr. Roswell Park, of Buffalo, sailed from Montreal for Europe, September 1, 1905. He will be absent about six weeks during which time he will attend the International Surgical Congress, of which he is a member. The meeting is to be held at Brussels, beginning September 18.

---

Dr. Hugh H. Young, of Baltimore, was the guest of Dr. H. E. Hayd for a few days in the early part of August.

---

Dr. Joseph D. Bryant, of New York, was chosen to deliver the annual oration on surgery before the American Medical Association at its meeting to be held at Boston next year.

---

Dr. Charles O'Reilly, who was superintendent of the Toronto general hospital for twenty-five years, resigned his office in May last. Early in June more than one hundred of his professional colleagues tendered Dr. O'Reilly a banquet at the Albany club, Toronto, which was presided over by Dr. Adam H. Wright. It was a memorable occasion and Dr. O'Reilly's service in advancing medical science and medical affairs in Toronto was the subject of just compliment. Dr. O'Reilly is in Europe for rest and recreation.

Dr. John F. Raub, of Washington, D. C., recently paid a visit to Buffalo and Niagara Falls. He was the guest of his son at the city of the cataract.

---

Dr. John J. Twohey, of Buffalo, attended the recent meeting of the American Medical Association at Portland, Oregon. In his tour to the Pacific coast and back he was accompanied by Mrs. Twohey.

---

Dr. W. Scott Renner, of Buffalo, sailed for Europe, August 15, contemplating an absence of about three months.

---

Dr. Edward T. Smith, of Buffalo, accompanied by Mrs. Smith, is making an extended European tour, during which he will visit the chief centers of medical learning.

